# Data concerning secondary structure and alpha-glucans-binding capacity of the *La*CBM26

**DOI:** 10.1016/j.dib.2018.11.056

**Published:** 2018-11-14

**Authors:** Silvia Armenta, Zaira Sánchez-Cuapio, Amelia Farrés, Karen Manoutcharian, Alejandra Hernandez-Santoyo, Sergio Sánchez, Romina Rodríguez-Sanoja

**Affiliations:** a Instituto de Investigaciones Biomédicas, Universidad Nacional Autónoma de México (UNAM), A.P. 70228, Ciudad Universitaria, Ciudad de México 04510, México; b Programa de Doctorado en Ciencias Bioquímicas, Universidad Nacional Autónoma de México (UNAM), A.P. 70228, Ciudad Universitaria, México DF 04510, Mexico; c Programa de Doctorado en Ciencias Biomédicas, Universidad Nacional Autónoma de México (UNAM), A.P. 70228, Ciudad Universitaria, México DF 04510, Mexico; d Facultad de Química, Universidad Nacional Autónoma de México, Circuito Exterior s/n Ciudad Universitaria, Ciudad de México 04510, México; e Instituto de Química, Universidad Nacional Autónoma de México, Circuito Exterior s/n Ciudad Universitaria, Ciudad de México 04510, México

**Keywords:** Carbohydrate-binding module, Starch, α-Glucans recognition

## Abstract

Carbohydrate-binding modules (CBMs) are auxiliary domains into glycoside-hydrolases that allow the interaction between the insoluble substrate and the solubilized enzyme, through hydrophobic, CH-π interactions and hydrogen bonds. Here, we present the data article related to the interaction of one *La*CBM26 and some mutated proteins with soluble α-glucans determined by enzyme-linked carbohydrate-binding assay, isothermal titration calorimetry (ITC), and affinity gel electrophoresis (AGE). The data of the behavior of proteins in presence and absence of substrate analyzed by circular dichroism CD and thermofluor are also presented. These results are complementary to the research article “The role of conserved non-aromatic residues in the *Lactobacillus amylovorus* α-amylase CBM26-starch interaction” (Armenta et al., 2019).

**Specifications table**

**Table:** 

Subject area	Biochemistry
More specific subject area	Protein characterization
Type of data	Graph, figure
How data was acquired	Multiskan FC (Thermo Scientific); J-710 spectropolarimeter (Jasco Inc., Easton, MD, USA); ITC_200_ (Malvern); CFX96 Real Time System C1000 Touch Thermal cycler (Bio-Rad Laboratories, Inc).
Data format	Analyzed
Experimental factors	Recombinant proteins were produced in *E. coli* and purified by immobilized metal affinity chromatography (ProBond Resin 46-0019, Invitrogen), followed by anion exchange chromatography (HiTrapTM DEAE FF 17–5055-01, GE Healthcare).
Experimental features	Enzyme-linked carbohydrate binding assay, CD in the far-UV, Fluorescence Thermal Shift Assay.
Data source location	Ciudad de México, México
Data accessibility	All data are shown within this article
Related research article	S. Armenta, Z. Sánchez-Cuapio, M.E. Munguia, N. Pulido, A. Farrés, K. Manoutcharian, A. Hernández-Santoyo, S. Moreno-Mendieta, S. Sánchez, R. Rodríguez-Sanoja, The role of non-aromatic residues in the *Lactoba*cillus amylovorus α-amylase CBM26-starch interaction, Int J Biol Macromol. 121 (2019) 829–838 [1].

**Value of the data**

• These data offer a system for the study of carbohydrate–protein interaction in an enzyme-linked carbohydrate-binding assay. The data obtained with this methodology agree with those observed with more used methodologies such as ITC and AGE.
• The data contribute to the study of proteins with atypical CD spectra that are rarely shown.
• Although active in binding, fluorescence signal plots (thermofluor) suggest an unfolding state of the LaCBM26 domain.

## 1 Data

**Fig. 1:**
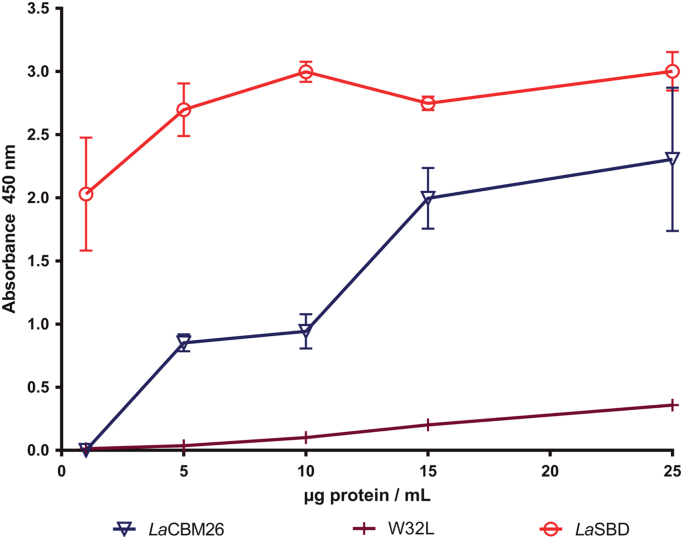
Protein interaction to immobilized soluble starch. *La*SBD: starch binding domain from *Lactobacillus amylovorus* α-amylase formed by five CBM26 in tandem. *La*CBM26: only one CBM26 domain of *La*SBD. W32L: CBM26 with a mutation in W32 that disables it to bind to starch or derived α-glucans. The error bars represent the standard deviation of triplicates.

**Fig. 2:**
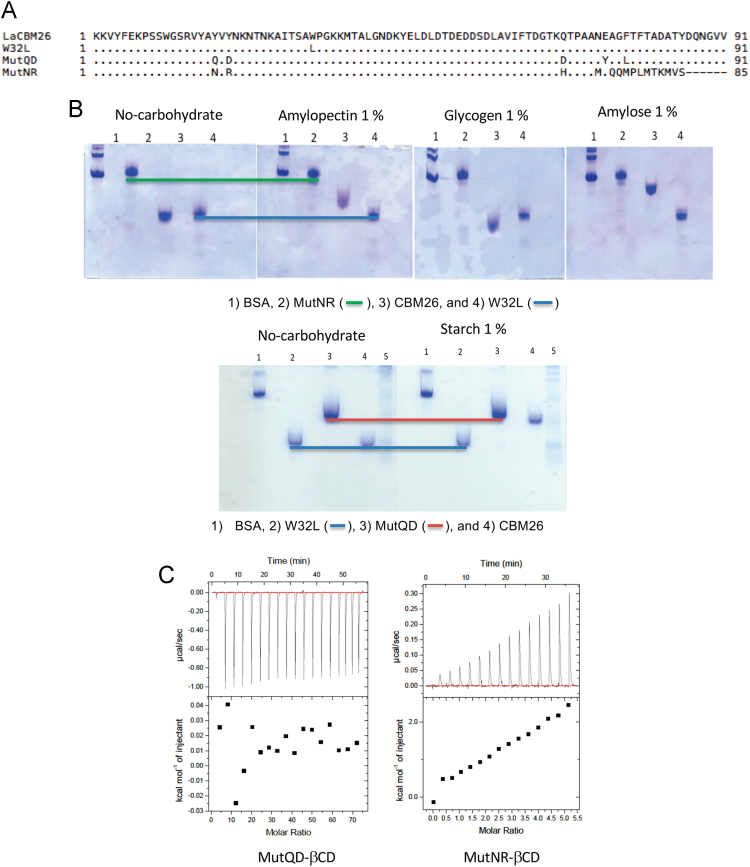
Glucan-binding of the *La*CBM26 mutated proteins. (A) Alignment of primary structure of the *La*CBM26 mutated proteins. (B) Affinity gel electrophoresis of representative variants. Image show the loss of interaction of the variants towards α-glucans. (C) Non-interaction observed by ITC calorimetry of selected variants.

**Fig. 3:**
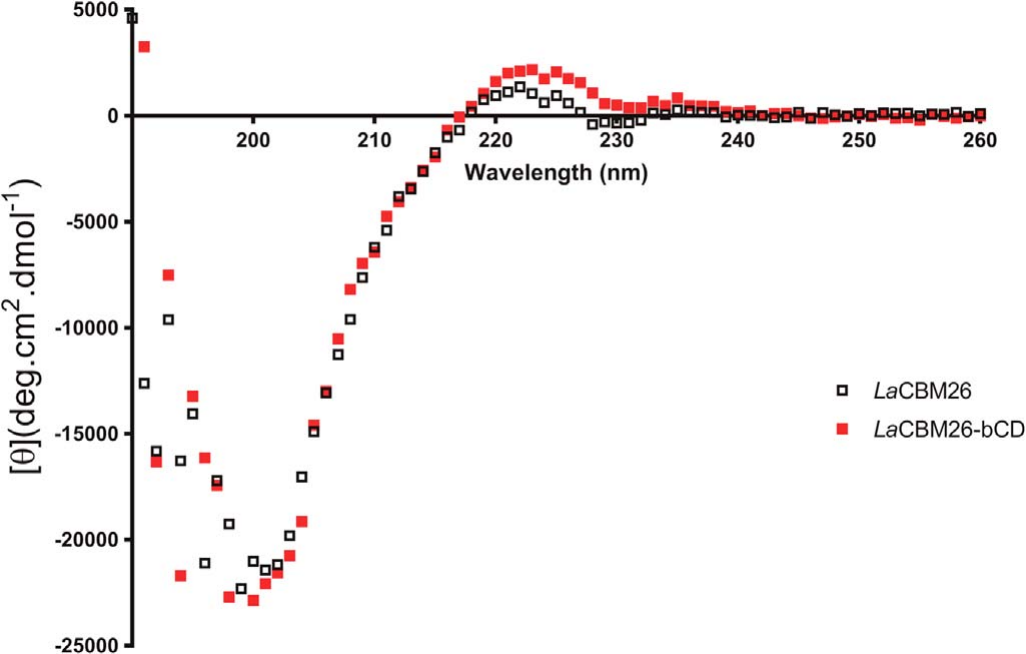
Thermofluor-based protein unfolding curves of the Starch Binding Domain from *Lactobacillus amylovorus* α-amylase (*La*SBD), and one CBM26 domain (*La*CBM26) with and without maltoheptose (M7). The data were acquired in 1 M sodium lactate/HCl buffer at the pH indicated in the legend.

**Fig. 4:**
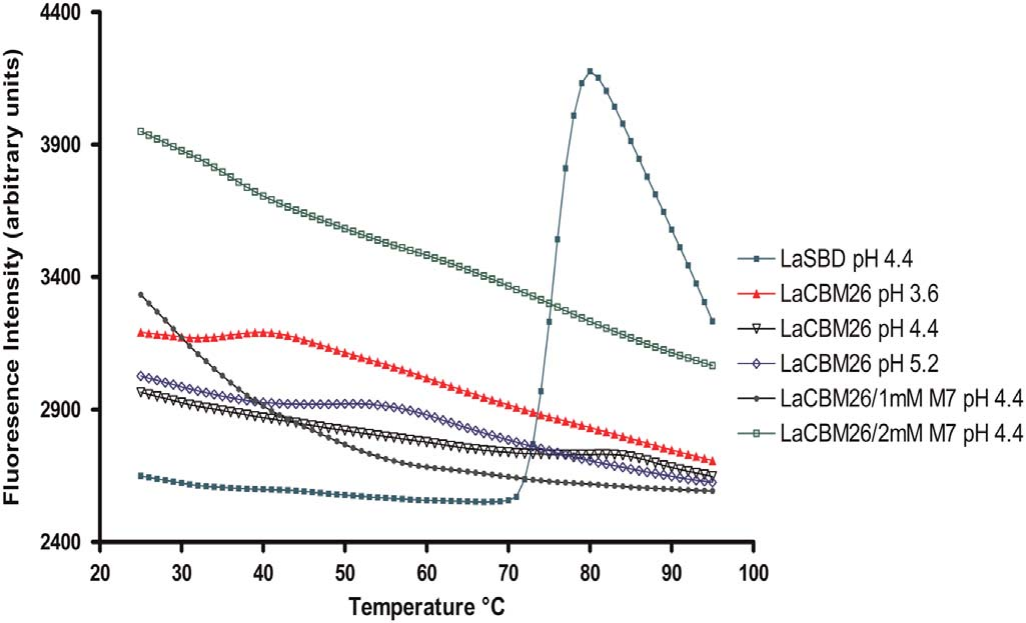
Far-UV CD spectra of *La*CBM26 with and without β-cyclodextrin (bCD). Spectra were obtained in 10 mM sodium phosphate buffer pH 7.4, at 20 °C.

In these data, the binding capacity of *Lactobacillus amylovorus* α-amylase Starch Binding Domain (*La*SBD) and one of the five identical modules (*La*CBM26) that constitutes the *La*SBD domain was determined by an adapted system of the enzyme-linked carbohydrate-binding assay (Fig. 1). In parallel, the preference of *La*CBM26 for linear substrates (glucose α-1,4) or branched at α-1,6 was investigated by affinity gel electrophoresis (Fig. 2).The thermostability of *La*CBM26 was estimated by measuring the fluorescence emission by thermally-induced protein melting [2]. Unlike the *La*SBD, *La*CBM26 seems to have exposed his hydrophobic patches and no change was observed in the presence of ligand (maltoheptaose) (Fig. 3). Similarly, there is no change in the circular dichroism spectrum due to the presence of the ligand (Fig. 4).

## 2 Experimental design, materials, and methods

### 2.1 Protein production and purification

*La*CBM26 and the mutated proteins were constructed and purified as previously described [1], [3].

### 2.2 Enzyme-linked carbohydrate-binding assay

Approximately 100 µL of 10% soluble starch (Prolabo Fontenay-sous-Bois, France) was used to coat 96-well microplates (Costar 3370) in phosphate-buffered saline (PBS) buffer, pH 7.4 for 22 h at room temperature (RT). Wells were washed three times with PBS, then blocked with 0.2% bovine serum albumin (BSA) in PBS-0.05%Tween 20 (PBS-5T) for 60 min at room temperature and wash once with PBS-5T.

The accessibility of the starch for the interaction was validated by measuring the adsorption of the *La*SBD, one *La*CBM26 and the null mutant protein W32 (CBM26 W32L) that does not interact with starch; at three different concentrations: 1 μg/mL, 10 μg/mL, and 25 μg/mL. Plates were incubated for 3 h at RT, followed by a wash step. The coated plate was then incubated 2 h at 37 °C with anti-His antibody (GeneTex) diluted 1:1,000 in PBS–BSA. Then, the plate was washed and incubated for 1.5 h at RT with anti-mouse IgG alkaline peroxidase (Invitrogen) diluted 1:5,000 in PBS–BSA. After washing, the plate was incubated for 30 min at RT with the FAST OPD substrate (SIGMA). Plates were read at 450 nm in a Multiskan FC (Thermo Scientific).

### 2.3 Affinity gel electrophoresis (AGE)

Affinity of mutated proteins and wild-type *La*CBM26 and derived mutated proteins for different carbohydrates was determined by affinity gel electrophoresis. The AGE method was performed with the Bio-Rad Mini-Protean III system (Bio-Rad, Richmond, CA). Purified proteins were separated in native gels containing 1% of potato starch (Prolabo, Fontenay-sous-Bois, France), amylose, amylopectin, pullulan, glycogen, cellulose, and birchwood xylan (all purchased from Sigma-Aldrich, St Louis Mo). Electrophoresis was run at 4 °C at 20 mA. Albumin was used as a negative, non-interacting control. Proteins were detected by staining with Coomassie brilliant blue [4].

### 2.4 Isothermal titration calorimetry (ITC)

The quantitative binding capacity of the wild-type *La*CBM26 and derived mutated proteins was determined by ITC using an ITC_200_ (Malvern) in 50 mM citrate-phosphate pH 5.5 at 25 °C as described in [1]. The data show no interaction between the protein and the carbohydrate; thus, no data analysis was done.

### 2.5 Thermal shift assay (Thermofluor)

To monitor protein unfolding, the hydrophobic fluorophore SYPRO orange was used. The thermal shift assay was conducted in the CFX96 Touch Real Time PCR Detection System (Bio-Rad Laboratories, Inc.). The buffer system used was 1 M sodium lactate/HCl buffer at different pH values (from 2.4 to 5.2), 1 µL of SYPRO orange (SYPRO orange in DMSO, Sigma-Aldrich; San Luis MO, USA) was added to 1 mL of *La*CBM26 10 µM. Ten microliters of the protein mix and 10 µL of the buffer solution were pipetted into the 96-well plate. The final volume of each reaction was 20 µL. The plate was heated from 25 to 95 °C with a heating rate of 1 °C/min. Fluorescence changes in the wells of the plate are monitored simultaneously using excitation and emission wavelengths of 492 and 516 nm, respectively.

### 2.6 Circular dichroism (CD)

CD spectroscopy was carried out at 20 °C on a J-710 spectropolarimeter (Jasco Inc., Easton, MD, USA) with purified proteins as described in [1]. The effect of the addition of the ligand on the secondary structure was checked by adding to *La*CBM26 13 µM, β-cyclodextrin to a final concentration of 39 µM. The raw data were converted to molar ellipticity ([θ]) using the formula: [θ] = θ*/(l x C x N*_*r*_*)* where θ is ellipticity in millidegrees, *l* is the cell path length in millimeters, *C* is the molar concentration of protein, and *N*_*r*_ is the number of residues [5].
